# Total hip arthroplasty in young adults, with focus on Perthes' disease and slipped capital femoral epiphysis: Follow-up of 540 subjects reported to the Norwegian Arthroplasty Register during 1987–2007

**DOI:** 10.3109/17453674.2011.641105

**Published:** 2012-04-24

**Authors:** Trude G Lehmann, Ingvild Ø Engesæter, Lene B Laborie, Stein Atle Lie, Karen Rosendahl, Lars B Engesæter

**Affiliations:** ^1^Department of Orthopaedic Surgery, Haukeland University Hospital; ^2^Institute of Surgical Science, University of Bergen; ^3^The Norwegian Arthroplasty Register, Haukeland University Hospital; ^4^Department of Radiology, Haukeland University Hospital; ^5^Uni Health, Uni Research, Bergen, Norway; Correspondence: trude.gundersen.lehmann@helse-bergen.no

## Abstract

**Background and purpose:**

Pediatric hip diseases account for 9% of all primary hip arthroplasties in the Norwegian Arthroplasty Register. We wanted to validate the diagnosis as reported to the register and to assess the quality of life of these patients after hip replacement.

**Patients and methods:**

540 patients accepted to participate in this follow-up study (634 hips). All were less than 40 years of age and had been reported to the Norwegian Arthroplasty Register as having undergone a primary total hip arthroplasty (THA) between 1987 and 2007. The underlying diagnosis, age at diagnosis, and type of treatment given prior to the hip replacement were recorded from the original hospital notes.

**Results:**

The diagnoses reported to the Norwegian Arthroplasty Register were confirmed to be correct in 91% of all cases (538/592). For the 94 hips that had been treated due to Perthes' disease or slipped capital femoral epiphysis (SCFE), the diagnosis was verified in 95% of cases (89/94). The corresponding proportion for inflammatory hip disease was 98% (137/140) and it was only 61% for primary osteoarthritis (19/31). The self reported quality of life (EQ-5D) was poorer for these young patients with THA than for persons in age-matched cohorts from Great Britain and Sweden, except for those with an underlying SCFE.

**Interpretation:**

The diagnoses reported to the Norwegian Arthroplasty Register as the underlying cause of THA were correct in 91% of cases. Individuals who undergo THA before the age of 40 have a reduced quality of life, except for those requiring a hip replacement because of SCFE.

Pediatric hip disorders such as developmental dysplasia of the hip (DDH), Perthes' disease, and slipped capital femoral epiphysis (SCFE) may lead to degenerative joint disease requiring a total hip arthroplasty (THA). According to data from the Norwegian Arthroplasty Register (NAR), pediatric hip disorders account for 9% of all primary hip arthroplasties (Norwegian Arthroplasty Register Annual Report 2010). Studies on the long-term outcome of Perthes' disease have indicated that the risk of later degenerative change varies according to age and the degree of involvement of the femoral head at presentation (Wiig et al. 2008). For SCFE, delayed diagnosis and treatment and the degree of residual deformity are associated with poorer functional outcome (Carney and Weinstein 1996, Gent and Clarke 2004). Only a few studies have addressed the quality of life of these 2 patient groups after hip replacement (Tellini et al. 2008, Wangen et al. 2008).

During the last 20–30 years, registries for THA have been established in all the Scandinavian countries. In Norway, an arthroplasty register has been running since 1987 (Havelin et al. 2000). Although reporting is not compulsory, the register has data on 98% of all hip replacements (Espehaug et al. 2006). However, little has been published on the validity of such registry data (Pedersen et al. 2004, Arthursson et al. 2005, Engesæter et al. 2011).

We therefore evaluated the accuracy of the diagnoses reported to the NAR for young adults. For patients with SCFE and Perthes' disease, we also determined the age at diagnosis and types of treatment given prior to THA, and also the quality of life following hip replacement.

## Patients and methods

### Patients

In this study we included patients born after January 1, 1967 (when the Medical Birth Registry of Norway was established) who had undergone THA and had been reported to the Norwegian Arthroplasty Register (NAR) during the period 1987–2007. 732 patients with 866 primary THAs were registered. 19 patients were excluded due to death or emigration. The remaining 713 patients were approached by letter and invited to complete 2 questionnaires on hip diagnosis and quality of life. After one reminder 578 (81%) responded, and of these, 540 patients (74% of the original cohort; corresponding to 634 hips) gave permission for further information on their hip disease to be collected from their medical records in the relevant hospital(s) (Figure) (Engesaeter et al. 2011).

**Figure:**
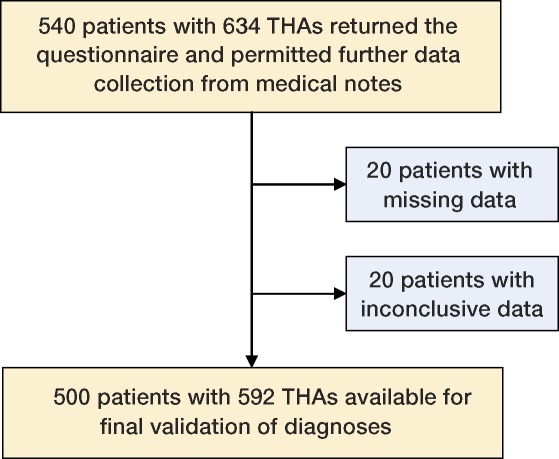
Flow of patients and THAs through the study.

### Questionnaires

The first questionnaire was custom-made, and included questions on age at diagnosis and whether or not they agreed to the diagnosis that had been reported to the NAR. If they disagreed on the diagnosis, they were asked to give the correct diagnosis.

The second questionnaire was EQ-5D, which is a standard health-related quality of life questionnaire that gives an EQ-5D index, where 0 is being dead and 100 is having the best possible health (Dolan 1997). An index of below 0 is ranked as a situation worse than death. We compared our findings with age-matched populations from Sweden and Great Britain (18–39 years) (Szende and Williams 2004).

### Collection of data from the medical records

For patients who agreed to the diagnosis recorded in the NAR, and who had been registered with a diagnosis of rheumatoid arthritis, ankylosing spondylitis (morbus Bechterew) or sequelae of a femoral neck fracture, we accepted the diagnosis as being correct (155 hips) without collecting further information from their medical records. For patients who had been registered as having primary osteoarthritis, hip dysplasia (DDH), Perthes' disease/slipped capital femoral epiphysis (SCFE), or “others”—and for patients who disagreed about the diagnosis recorded—further information was obtained from the hospital notes (479 hips). The medical notes were searched for information on age at the time of diagnosis and all the treatment given. Original radiographs were unavailable for many of the patients, due to Norwegian legislation which states that radiographs do not need to be stored for more than 10 years after the last contact with the patient. The 634 hip replacements were performed in 48 hospitals. Data from the medical records were either collected directly from the 14 hospitals that performed 5 or more THAs or were received by post from the remaining 34 hospitals. Data on 22 hips (in 20 patients) were missing, and data on 20 hips (in 20 patients) were inconclusive, leaving 500 patients (68% of the original cohort) with 592 THAs for further analysis (Figure 1). Patients reported to have the diagnoses sequelae of dysplasia or sequelae of dysplasia with luxation (dislocated at the time of THA) were pooled into one group (sequelae of DDH) for further analysis.

For diagnoses that were found to be incorrect after validation, we collected the original form submitted to the NAR and decided whether an incorrect diagnosis had been reported by the surgeon or whether there had been an error during the registration process.

### Incidence of SCFE in Norway

The incidence of Perthes' disease in Norway has been reported to be 9.2 per 10^5^ (Wiig et al. 2008). There has been no similar study for SCFE, and an incidence was therefore calculated based on data from the Norwegian Patient Register (NPR). This is a mandatory, national registry to which all hospitals report regarding diagnoses and operation codes when patients are discharged. The incidence of SCFE for subjects less than 16 years of age during the period 2000–2009 was calculated based on original data that had been reported concerning the annual number of hips diagnosed with SCFE (ICD-10 code M930), with a corresponding primary operation code. This was divided by the total number of individuals under 16 years of age in Norway during the same period. These data were received from Statistics Norway (www.ssb.no). To obtain the annual incidence of patients operated for SCFE, the mean annual number of operated hips was adjusted down based on the assumption that about 20–30% of patients with SCFE have bilateral operations (Hägglund et al. 1984, Loder 1996, Lehmann et al. 2011).

### The Norwegian Arthroplasty Register

The registration form is filled in by the surgeon immediately after the operation and includes information on date of surgery, underlying hip disorder classified into 1 of 9 categories (Table 1), the type of surgery, and whether it was primary surgery or a re-operation (Havelin et al. 2000). The diagnoses of Perthes' disease and SCFE are, however, pooled in the registration form as 1 common option.

**Table 1.** Baseline characteristics of attendees and non-attendees for the cohort and for subjects with THA secondary to Perthes'/SCFE reported to the NAR

	Attendees	95% CI	Non-attendees	95% CI	p-value
Number of patients	500		232		
Male, n (%)	218 (44%)	39–48	127 (55%)	48–61	0.008
Age at THA, mean	28.7	28–29	28.3	27–29	0.5
Type of underlying diagnosis, n (%)					
Primary osteoarthrosis	28 (5.6%)	3.5–7.7	19 (8.3%)	4.6–12	0.2
JIA / RA	81 (16.1%)	13–20	25 (11%)	6.7–15	0.06
Sequelae of femoral neck fracture	26 (5.2%)	3.2–7.2	19 (8.3%)	4.6–12	0.1
Sequelae of DDH	129 (25.7%)	22–30	44(19%)	14–24	0.052
Sequelae of Perthes'/ SCFE	91 (18.1%)	15–22	42 (18%)	13–23	1.0
Ankylosing spondylitis	20 (4.0%)	2.2–5.8	8 (3.5%)	1.1–5.8	0.7
Acute femoral neck fracture	2 (0.4%)	–1.6 to 2.4	0		0.3
Others	126 (25.1%)	21–29	73 (32%)	25–38	0.06
Perthes' disease/SCFE	91		42		
Male, n (%)	61 (67%)	57–77	32 (76%)	9.3–18	0.3
Age at THA, mean	27.5	26–29	25.8	24–28	0.2

JIA: juvenile idiopathic arthritis; RA: rheumatoid arthritis; DDH: developmental dysplasia of the hip; SCFE: slipped capital femoral epiphysis.

### Ethics

The study was approved by the Regional Ethics Committee for Medical and Health Research, reg. number 238.03, and written informed consent was obtained from all the participants.

### Statistics

The data are summarized using mean (range). Means were compared using independent-samples t-test. The approach of Welch was used when equal variance was assumed, based on Levene's test for equality. Chi-square tests were used to compare attendees with non-attendees. Both hips were used when validating the correctness of reported diagnoses. There were no differences in the results when only 1 hip from each patient was used. Analyses of baseline characteristics, prior treatment, and quality of life were done on the patients. All analyses were performed with the SPSS software version 17.0.

## Results

500 patients (344 females, 592 THAs) were included. Except for more females attending (p = 0.008), there were no statistically significant differences in baseline characteristics between the 500 attendees and the 213 non-attendees (Table 1). Mean age at the time of hip replacement was 29 (12–41) years, with no significant differences between the sexes. Mean age at follow-up was 35 (17–41) years.

538 of the 592 registry-based diagnoses were compared to questionnaires/medical records and judged to be correct in 91% of cases (95% CI: 83–99) (Table 2).

**Table 2.** Validation of diagnoses reported to the Norwegian Arthroplasty Register based on the original medical records and patient self-reporting

Diagnosis as reported to the NAR	No. of THAs	Correct diagnosis	95% CI
Primary osteoarthrosis	31	19 (61%)	44–79
JIA / RA	112	109 (97%)	94–100
Sequelae of femoral neck fracture	26	25 (96%)	87–104
Sequelae of DDH	150	132 (88%)	83–93
Sequelae of Perthes'/SCFE	94	89 (95%)	90–99
Ankylosing spondylitis (morbus Bechterew)	28	28 (100%)	100–100
Acute fracture of the femoral neck	2	2 (100%)	100–100
Others, specified	145	134 (92%)	84–101
Missing diagnosis	4	0	0–0
Total	592	538	

Abbreviations: See Table 1.

41% (240/592) of the THAs had been performed due to hip dysplasia (DDH) (142 hips), Perthes' disease (72 hips), or SCFE (29 hips). For 3 patients, the diagnoses of both DDH and Perthes' disease had been reported and had also been suggested in the medical notes. For the purposes of this study, these patients were included in the Perthes' disease group.

Of the 240 THAs performed due to pediatric hip disorders, 221 (92%) were correctly registered in the NAR (Table 2). 18 hips that had been incorrectly reported as hip dysplasia were validated to be Perthes' disease (5 hips), SCFE (1 hip), or other specified diagnoses (12 hips). 13 hips that had been incorrectly reported as primary osteoarthritis were re-diagnosed as hip dysplasia or other specified pathologies. 98% of hips (137/140) that were operated due to rheumatoid arthritis or ankylosing spondylithis had been correctly diagnosed initially (Table 2).

43 of the 54 incorrect diagnoses registered in the NAR were due to mistakes made by the surgeons in filling in the forms. However, for 8 hips reported as “other specified diagnosis”, the surgeon had also noted the correct diagnosis but this had not been registered correctly in the NAR (4 sequelae of femoral neck fracture, 3 SCFE, and 1 juvenile idiopathic arthritis/RA). 3 hips had been reported correctly on the form by the surgeon but had been wrongly registered by the secretary at the NAR.

101 of 500 subjects (20%) underwent THA due to Perthes' disease (72 patients, 52 males) or SCFE (29 patients, 16 females) (Table 3). None of these had had bilateral THA. For the 72 patients with Perthes' disease, information on treatment prior to the THA was available for 44. 24 patients had undergone surgery, while 20 had only received nonoperative treatment. For patients with SCFE, age at diagnosis and operation was 13 (10–15) years. 1 patient presented with symptoms at the age of 24 years and underwent THA 7 years later.

**Table 3.** Frequency of different hip disorders after validation. In 5 hips, 2 diagnosis were likely: 2 hips showed RA and DDH (same patient) and 3 hips showed DDH and Perthes' disease

Diagnosis	Correct numbers after validation (hips)
Primary arthrosis	18
JIA / RA	110
Sequelae of femoral neck fracture	31
Sequelae of DDH	142
Sequelae of Perthes' disease	72
Sequelae of SCFE	29
Ankylosing spondylitis	30
Acute fracture of the femoral neck	2
Others, specified	162
Missing diagnosis	0

Abbreviations: See Table 1.

### Quality of life, EQ-5D

The mean EQ-5D index score for all subjects (500 patients) was 71 (8–100), 73 for males and 70 for females (p = 0.2), which was lower than that reported for an age-matched cohort in Sweden (89) and the UK (86) (p < 0.001). The mean score for those who underwent THA due to SCFE was significantly higher than that reported for those with Perthes' (81 vs. 74; p = 0.04) or hip dysplasia (81 vs. 69; p = 0.008) (Table 4). The score for those operated because of SCFE was similar to that reported for an age-matched cohort in the UK (p = 0.13), but it was lower than that reported for an age-matched population in Sweden (p = 0.03).

**Table 4.** EQ-5D index for patients with different diagnoses

Diagnosis	EQ-5D scores **^a^**	(95% CI)
Primary osteoarthrosis	73	61–85
JIA / RA	66	61–71
Sequelae of femoral neck fracture	68	59–77
Sequelae of DDH	69	65–73
Sequelae of Perthes	74	70–79
Sequelae of SCFE	81	75–87
Ankylosing spondylithis	73	64–82
Acute fracture of the femoral neck **^b^**	44	36–52
Others, specified	74	70–78
All diagnoses	71	69–73

**^a^** 0 = worse imaginable health; 100 = best possible health.

**^b^** Only 2 patients were operated due to acute fracture of the femoral neck.

### Incidence of SCFE

The annual number of hips operated for SCFE that were reported to the NPR during the period 2000–2009 varied from 29 to 46, with a mean of 38, giving an annual incidence of diagnosed hips with SCFE of 4 per 10^5^ for children below the age of 16 years. When adjusting for bilaterality, this gave an annual incidence of patients with SCFE of about 3 per 10^5^.

## Discussion

We found that the underlying cause of total hip replacement was correctly reported to the NAR in 91% of all subjects less than 40 years of age and in 95% of patients operated due to Perthes' disease or SCFE. Except for those operated due to an underlying SCFE, quality of life as assessed by the EQ-5D index was poorer than in age-matched cohorts.

The strengths of our study include the high number of participants and the collection of additional data from the medical records. Except for gender, there were no statistically significant differences between the baseline data of the 213 non-attendees and those of the attendees. This was not unexpected, since females are more liable to respond to surveys (Hill et al. 1997). Thus, there is little reason to believe that our cohort was flawed by selection bias.Our findings regarding validation of diagnoses compare favorably with a study from the Danish Hip Arthroplasty Register involving 459 patients (Pedersen et al. 2004). After having reviewed the medical records and preoperative radiographs, these authors found that a reported diagnosis had a positive predictive value of 84%. The outcome most probably reflects difficulties in assessing an underlying diagnosis in older age groups, as secondary degenerative changes tend to obscure underlying pathologies.

The observation that primary osteoarthritis was the diagnosis that was most commonly incorrectly reported was not unexpected, since severe arthritis warranting a THA at this young age would tend to obscure an underlying diagnosis such as DDH, Perthes' disease, or SCFE (Murray 1965).

Children with Perthes' disease had their diagnosis at 7 years of age; one third of them had undergone surgical treatment and slightly less than one third had had nonoperative treatment alone. According to the medical notes, the remainder had received no treatment at all—although 17 of these patients reported otherwise in the questionnaire. This controversy may be due in part to inaccurate medical notes, recall bias, or both. For those with SCFE, age at diagnosis was slightly higher—around 12 years—and all but 1 had had prompt surgery at the time of diagnosis.

The initial treatment of Perthes' disease depends on age and the severity of femoral head necrosis (Wiig et al. 2008). For SCFE, the standard treatment is operative stabilization of the femoral epiphysis (Loder et al. 2008), aiming at prevention or delay of degenerative change (Carney et al. 1991). In the present study, all but 1 of the patients with SCFE and one third of those with Perthes' disease had hip-preserving surgery as adolescents. Since most of the initial radiographs were unavailable, we could not determine whether those receiving surgery for Perthes' disease were more severely affected than those who were treated nonoperatively. Likewise, we were unable to determine the degree of slip prior to initial surgery.

Calculation of the annual incidence of SCFE in children less than 16 years was based on the NPR. Diagnoses of SCFE in the NPR are not validated, but a study from Arthurson et al. (2005) reported a difference of only 3.4% in data reported to this mandatory, national register compared to data reported to the NAR from a single hospital. All hospitals are obliged to report their patients to this administrative register, and there is little reason to believe that patients with SCFE were under- or over-represented. The NPR contains information on the number of operated hips only, not to the number of operated children. However, in a recent study, we showed that 30% of children with SCFE suffer bilateral involvement (Lehmann et al. 2011), providing a ratio on which to base the estimated incidence. This number is in accordance with that reported by other authors (Hägglund et al. 1984, Loder 1996). Moreover, none of the hospitals performed prophylactic fixation of the contralateral hip during the study period. The annual incidence of 3 per 10^5^ also compares well with other studies (Loder et al. 2000, Krauspe et al 2004). In comparison, the incidence of Perthes' disease was found to be 9 per 10^5^ in a large, randomized national trial (Wiig et al. 2008).

When comparing the number of operated prosthesis in SCFE or Perthes'disease with the incidences of the diseases (3 per 10^5^ for SCFE and 9 per 10^5^ for Perthes' disease), we can see that the risk of undergoing THA was about the same for the 2 diseases.

Of those patients who underwent THA as a result of SCFE, more than half of them (16 of 29) were females. This was rather surprising since SCFE is seen more frequently in males (2:1) (Loder et al. 2000, Gholve et al. 2009). One explanation may be that girls suffer a more severe slip than boys. Again, due to the unavailability of the initial radiographs, we could not investigate this in detail. One could speculate that there is a diagnostic delay leading to a more severe slip in girls, since doctors are more prone to consider SCFE as a possible diagnosis in males. A previous study of 67 patients with SCFE from our institution supports this, in that the female patients had almost 2 months longer duration of symptoms and nearly two-thirds had a moderate or severe slip compared to one third of the boys (Lehmann et al. 2011). Longer duration of symptoms is known to increase the severity of the slip (Loder et al. 2006).

In this study, we found that the quality of life after THA is reduced for patients below 40 years of age as compared to healthy age-matched controls. This has also been demonstrated by Wangen et al. (2008) in a study of 49 patients aged 30 or less. They found a mean EQ-5D index of 68 as compared to 71 in our study. In comparison, indices from age-matched Swedish or British cohorts have been reported as being 85–90 (Szende and Williams 2004). When subdivided into different diagnoses, we found that patients who had been operated due to SCFE had a better quality of life than the other groups. The reasons for this are unclear, but one explanation may be that the femoral head alone, and not the acetabulum, is involved in the underlying disease. In another study from the NAR (Engesæter et al. 2003), it was found that revision rates for patients requiring THA due to Perthes' disease/SCFE were lower than for other diagnoses, but since both Perthes' disease and SCFE are reported under the same tick box in the registration form, it was not possible to ascertain whether SCFEs or the Perthes' disease caused the favorable results in that study. In the next revision of the NAR form, the diagnosis of Perthes' disease and SCFE will be registered separately.

In conclusion, data held in the NAR on the underling diagnosis for THA in young adults was of high quality, with 91% of the diagnoses being correctly reported. THA patients had a poorer quality of life than those in age-matched cohorts in Sweden and the UK, except for those who underwent THA as a result of SCFE.
